# Supplementary material for: Risk assessment of temporary pacing for cardiac arrest after cardiopulmonary bypass-assisted cardiovascular surgery: A case-control study
Source: PLoS One. 2025 May 19;20(5):e0323795. doi: 10.1371/journal.pone.0323795 (PMC12088002; doi:10.1371/journal.pone.0323795)
Supplement: S1 Table — (DOCX) [file pone.0323795.s001.docx]

**S1 Table. The initial multiple logistic regression.^#^**

| **Characteristic** | **OR (95%CI)** | **P value** |
| --- | --- | --- |
| **Sex** |  |  |
| Male | Ref. | Ref. |
| Female | 1.147 (0.7578-1.740) | P=0.5168 |
| **Age (per year)** | 1.039 (1.022-1.058) | P<0.0001 |
| **BMI (per kg·m^-2^)** | 1.000 (0.9404-1.062) | P=0.9952 |
| **Preoperative rhythm** |  |  |
| Sinus rhythm | Ref. | Ref. |
| Atrial fibrillation | 3.638 (2.159-6.059) | P<0.0001 |
| **Operation** |  |  |
| CABG | Ref. | Ref. |
| MVR | 5.224 (1.399-34.06) | P=0.0327 |
| AVR | 3.857 (0.8973-26.47) | P=0.0992 |
| DVR | 5.276 (1.344-35.16) | P=0.0357 |
| MVR+TVP | 7.672 (2.022-50.41) | P=0.0090 |
| MVP | 4.695 (0.9283-34.57) | P=0.0775 |
| CABG+MVR | 5.277 (1.132-37.47) | P=0.0495 |
| DVR+TVP | 2.503 (0.3815-20.31) | P=0.3382 |
| ASD closure | 6.033 (0.2672-68.21) | P=0.1561 |
| Other | 3.948 (1.132-24.97) | P=0.0669 |
| **Ablation** |  |  |
| No | Ref. | Ref. |
| Yes | 0.8938 (0.4895-1.613) | P=0.7112 |
| **Pump** |  |  |
| Occlusive | Ref. | Ref. |
| Centrifugal | 1.216 (0.1817-4.733) | P=0.8056 |
| **Cardioplegia type** |  |  |
| Crystal | Ref. | Ref. |
| Cold blood | 0.9800 (0.3962-2.706) | P=0.9667 |
| **Cardioplegia volume (per ml)** | 1.000 (0.9998-1.000) | P=0.6570 |
| **Hypothermia** |  |  |
| Mild | Ref. | Ref. |
| Moderate | 0.7650 (0.4223-1.318) | P=0.3539 |
| Deep | 1.156 (0.1254-6.836) | P=0.8883 |
| **Circulation** |  |  |
| Normal | Ref. | Ref. |
| Arrested or low-flow | 0.5711 (0.07470-5.439) | P=0.6139 |
| **CPB time (per min)** | 1.006 (1.001-1.012) | P=0.0173 |
| **Aortic clamping time (per min)** | 0.9977 (0.9892-1.006) | P=0.5928 |

#. Abbreviation: ASD, atrial septal defect; AVR, aortic valve replacement; BMI, body mass index; CABG, coronary artery bypass grafting; CI, confidence interval; CPB, cardiopulmonary bypass; DVR, double valve replacement; MVP, mitral valvuloplasty; MVR, mitral valve replacement; OR, odds ratio; TVP, tricuspid valvuloplasty.
