# Supplementary material for: Risk assessment of temporary pacing for cardiac arrest after cardiopulmonary bypass-assisted cardiovascular surgery: A case-control study
Source: PLoS One. 2025 May 19;20(5):e0323795. doi: 10.1371/journal.pone.0323795 (PMC12088002; doi:10.1371/journal.pone.0323795)
Supplement: S2 Table — (DOCX) [file pone.0323795.s002.docx]

**S2 Table. The multiple logistic regression with squared continuous variables.^#^**

| **Characteristic** | **OR** | **95%CI lower limit** | **95%CI upper limit** | **P value** | **Significance** |
| --- | --- | --- | --- | --- | --- |
| **Sex** |  |  |  |  |  |
| Male | Ref. | | | | |
| Female | 1.1180 | 0.7406 | 1.6920 | 0.5948 | ns |
| **Age^2^ (per year)** | 1.0000 | 1.0000 | 1.0000 | <0.0001 | **** |
| **BMI^2^ (per kg·m^-2^)** | 1.0000 | 0.9989 | 1.0010 | 0.7839 | ns |
| **Preoperative rhythm** |  |  |  |  |  |
| Sinus rhythm | Ref. | | | | |
| Atrial fibrillation | 3.7190 | 2.2070 | 6.1900 | <0.0001 | **** |
| **Operation** |  |  |  |  |  |
| CABG | Ref. | | | | |
| MVR | 4.5870 | 1.2340 | 29.8200 | 0.0485 | * |
| AVR | 3.4200 | 0.8003 | 23.3800 | 0.1319 | ns |
| DVR | 2.4200 | 0.3721 | 19.5000 | 0.3538 | * |
| MVR+TVP | 6.9880 | 1.8530 | 45.7800 | 0.0125 | * |
| MVP | 4.2440 | 0.8438 | 31.1300 | 0.0980 | ns |
| CABG+MVR | 5.3230 | 1.1460 | 37.7200 | 0.0480 | * |
| DVR+TVP | 2.4200 | 0.3721 | 19.5000 | 0.3538 | ns |
| ASD closure | 4.6550 | 0.2079 | 51.9100 | 0.2222 | ns |
| Other | 3.6920 | 1.0600 | 23.3400 | 0.0811 | ns |
| **Ablation** |  |  |  |  |  |
| No | Ref. | | | | |
| Yes | 0.9364 | 0.5150 | 1.6830 | 0.8272 | ns |
| **Pump** |  |  |  |  |  |
| Occlusive | Ref. | | | | |
| Centrifugal | 1.2190 | 0.1834 | 4.6800 | 0.8016 | ns |
| **Cardioplegia type** |  |  |  |  |  |
| Crystal | Ref. | | | | |
| Cold blood | 0.8986 | 0.3757 | 2.4220 | 0.8200 | ns |
| **Cardioplegia volume^2^ (per ml)** | 1.0000 | 1.0000 | 1.0000 | 0.9444 | ns |
| **Hypothermia** |  |  |  |  |  |
| Mild | Ref. | | | | |
| Moderate | 0.8037 | 0.4464 | 1.3750 | 0.4439 | ns |
| Deep | 1.0980 | 0.1140 | 7.2110 | 0.9323 | ns |
| **Circulation** |  |  |  |  |  |
| Normal | Ref. | | | | |
| Arrested or low-flow | 0.6909 | 0.0832 | 6.6600 | 0.7502 | ns |
| **CPB time^2^ (per min)** | 1.0000 | 1.0000 | 1.0000 | 0.0578 | ns |
| **Aortic clamping time^2^ (per min)** | 1.0000 | 1.0000 | 1.0000 | 0.8535 | ns |

#. Abbreviation: ASD, atrial septal defect; AVR, aortic valve replacement; BMI, body mass index; CABG, coronary artery bypass grafting; CI, confidence interval; CPB, cardiopulmonary bypass; DVR, double valve replacement; MVP, mitral valvuloplasty; MVR, mitral valve replacement; ns, no significance; OR, odds ratio; TVP, tricuspid valvuloplasty.
