# Supplementary material for: Risk assessment of temporary pacing for cardiac arrest after cardiopulmonary bypass-assisted cardiovascular surgery: A case-control study
Source: PLoS One. 2025 May 19;20(5):e0323795. doi: 10.1371/journal.pone.0323795 (PMC12088002; doi:10.1371/journal.pone.0323795)
Supplement: S3 Table — (DOCX) [file pone.0323795.s003.docx]

**S3 Table. The multiple logistic regression with cubic continuous variables.^#^**

| **Characteristic** | **OR** | **95%CI lower limit** | **95%CI upper limit** | **P value** | **Significance** |
| --- | --- | --- | --- | --- | --- |
| **Sex** |  |  |  |  |  |
| Male | Ref. | | | | |
| Female | 1.1040 | 0.7325 | 1.6680 | 0.6354 | ns |
| **Age^3^ (per year)** | 1.0000 | 1.0000 | 1.0000 | <0.0001 | **** |
| **BMI^3^ (per kg·m^-2^)** | 1.0000 | 1.0000 | 1.0000 | 0.6627 | ns |
| **Preoperative rhythm** |  |  |  |  |  |
| Sinus rhythm | Ref. | | | | |
| Atrial fibrillation | 3.7650 | 2.2330 | 6.2700 | <0.0001 | **** |
| **Operation** |  |  |  |  |  |
| CABG | Ref. | | | | |
| MVR | 4.2730 | 1.1510 | 27.7700 | 0.0598 | ns |
| AVR | 3.2130 | 0.7530 | 21.9500 | 0.1523 | ns |
| DVR | 4.8600 | 1.2520 | 32.1600 | 0.0445 | * |
| MVR+TVP | 6.5890 | 1.7510 | 43.1100 | 0.0152 | * |
| MVP | 4.0650 | 0.8124 | 29.7400 | 0.1075 | ns |
| CABG+MVR | 5.4290 | 1.1740 | 38.4100 | 0.0450 | * |
| DVR+TVP | 2.4220 | 0.3743 | 19.4300 | 0.3521 | ns |
| ASD closure | 3.8480 | 0.1726 | 42.6300 | 0.2835 | ns |
| Other | 3.5770 | 1.0270 | 22.6100 | 0.0887 | ns |
| **Ablation** |  |  |  |  |  |
| No | Ref. | | | | |
| Yes | 0.9670 | 0.5327 | 1.7370 | 0.9112 | ns |
| **Pump** |  |  |  |  |  |
| Occlusive | Ref. | | | | |
| Centrifugal | 1.1850 | 0.1791 | 4.5060 | 0.8287 | ns |
| **Cardioplegia type** |  |  |  |  |  |
| Crystal | Ref. | | | | |
| Cold blood | 0.8452 | 0.3580 | 2.2590 | 0.7174 | ns |
| **Cardioplegia volume^3^ (per ml)** | 1.0000 | 1.0000 | 1.0000 | 0.9749 | ns |
| **Hypothermia** |  |  |  |  |  |
| Mild | Ref. | | | | |
| Moderate | 0.8314 | 0.4643 | 1.4130 | 0.5133 | ns |
| Deep | 1.0530 | 0.1035 | 7.3990 | 0.9642 | ns |
| **Circulation** |  |  |  |  |  |
| Normal | Ref. | | | | |
| Arrested or low-flow | 0.7742 | 0.0888 | 7.6110 | 0.8310 | ns |
| **CPB time^3^ (per min)** | 1.0000 | 1.0000 | 1.0000 | 0.1082 | ns |
| **Aortic clamping time^3^ (per min)** | 1.0000 | 1.0000 | 1.0000 | 0.5601 | ns |

#. Abbreviation: ASD, atrial septal defect; AVR, aortic valve replacement; BMI, body mass index; CABG, coronary artery bypass grafting; CI, confidence interval; CPB, cardiopulmonary bypass; DVR, double valve replacement; MVP, mitral valvuloplasty; MVR, mitral valve replacement; ns, no significance; OR, odds ratio; TVP, tricuspid valvuloplasty.
