# Supplementary material for: Risk assessment of temporary pacing for cardiac arrest after cardiopulmonary bypass-assisted cardiovascular surgery: A case-control study
Source: PLoS One. 2025 May 19;20(5):e0323795. doi: 10.1371/journal.pone.0323795 (PMC12088002; doi:10.1371/journal.pone.0323795)
Supplement: S4 Table — (DOCX) [file pone.0323795.s004.docx]

**S4 Table. The multiple logistic regression with square-rooted continuous variables.^#^**

| **Characteristic** | **OR** | **95%CI lower limit** | **95%CI upper limit** | **P value** | **Significance** |
| --- | --- | --- | --- | --- | --- |
| **Sex** |  |  |  |  |  |
| Male | Ref. | | | | |
| Female | 1.1650 | 0.7689 | 1.7680 | 0.4716 | ns |
| **Age^1/2^ (per year)** | 1.7730 | 1.3770 | 2.3300 | <0.0001 | **** |
| **BMI^1/2^ (per kg·m^-2^)** | 0.9539 | 0.5306 | 1.7020 | 0.8738 | ns |
| **Preoperative rhythm** |  |  |  |  |  |
| Sinus rhythm | Ref. | | | | |
| Atrial fibrillation | 3.5830 | 2.1250 | 5.9720 | <0.0001 | **** |
| **Operation** |  |  |  |  |  |
| CABG | Ref. | | | | |
| MVR | 5.7010 | 1.5180 | 37.2700 | 0.0250 | * |
| AVR | 4.1970 | 0.9706 | 28.9200 | 0.0808 | ns |
| DVR | 5.5440 | 1.4050 | 37.0600 | 0.0311 | * |
| MVR+TVP | 8.1130 | 2.1280 | 53.4400 | 0.0075 | ** |
| MVP | 5.0190 | 0.9896 | 37.0400 | 0.0660 | ns |
| CABG+MVR | 5.3650 | 1.1510 | 38.1100 | 0.0474 | * |
| DVR+TVP | 2.6000 | 0.3951 | 21.1600 | 0.3194 | ns |
| ASD closure | 6.8870 | 0.3031 | 78.6700 | 0.1294 | ns |
| Other | 4.1570 | 1.1910 | 26.3100 | 0.0574 | ns |
| **Ablation** |  |  |  |  |  |
| No | Ref. | | | | |
| Yes | 0.8757 | 0.4782 | 1.5840 | 0.6632 | ns |
| **Pump** |  |  |  |  |  |
| Occlusive | Ref. | | | | |
| Centrifugal | 1.1910 | 0.1778 | 4.6470 | 0.8260 | ns |
| **Cardioplegia type** |  |  |  |  |  |
| Crystal | Ref. | | | | |
| Cold blood | 1.0350 | 0.4130 | 2.8880 | 0.9434 | ns |
| **Cardioplegia volume^1/2^ (per ml)** | 1.0070 | 0.9890 | 1.0240 | 0.4575 | ns |
| **Hypothermia** |  |  |  |  |  |
| Mild | Ref. | | | | |
| Moderate | 0.7472 | 0.4124 | 1.2890 | 0.3139 | ns |
| Deep | 1.1520 | 0.1242 | 6.5960 | 0.8891 | ns |
| **Circulation** |  |  |  |  |  |
| Normal | Ref. | | | | |
| Arrested or low-flow | 0.5318 | 0.0719 | 5.1020 | 0.5640 | ns |
| **CPB time^1/2^ (per min)** | 1.2200 | 1.0510 | 1.4110 | 0.0077 | ** |
| **Aortic clamping time^1/2^ (per min)** | 0.9168 | 0.7693 | 1.0920 | 0.3295 | ns |

#. Abbreviation: ASD, atrial septal defect; AVR, aortic valve replacement; BMI, body mass index; CABG, coronary artery bypass grafting; CI, confidence interval; CPB, cardiopulmonary bypass; DVR, double valve replacement; MVP, mitral valvuloplasty; MVR, mitral valve replacement; ns, no significance; OR, odds ratio; TVP, tricuspid valvuloplasty.
