# Supplementary material for: Risk assessment of temporary pacing for cardiac arrest after cardiopulmonary bypass-assisted cardiovascular surgery: A case-control study
Source: PLoS One. 2025 May 19;20(5):e0323795. doi: 10.1371/journal.pone.0323795 (PMC12088002; doi:10.1371/journal.pone.0323795)
Supplement: S5 Table — (DOCX) [file pone.0323795.s005.docx]

**S5 Table. The multiple logistic regression without outliers identified by the ROUT method.^#^**

| **Characteristic** | **OR** | **95%CI lower limit** | **95%CI upper limit** | **P value** | **Significance** |
| --- | --- | --- | --- | --- | --- |
| **Sex** |  |  |  |  |  |
| Male | Ref. | | | | |
| Female | 1.1080 | 0.7122 | 1.7280 | 0.6493 | ns |
| **Age (per year)** | 1.0390 | 1.0200 | 1.0590 | <0.0001 | **** |
| **BMI (per kg·m^-2^)** | 1.0140 | 0.9495 | 1.0810 | 0.6778 | ns |
| **Preoperative rhythm** |  |  |  |  |  |
| Sinus rhythm | Ref. | | | | |
| Atrial fibrillation | 3.8360 | 2.1930 | 6.6320 | <0.0001 | **** |
| **Operation** |  |  |  |  |  |
| CABG | Ref. | | | | |
| MVR | 6.1810 | 1.5380 | 42.0900 | 0.0238 | * |
| AVR | 5.0650 | 1.1190 | 36.1000 | 0.0546 | ns |
| DVR | 6.3770 | 1.4600 | 45.3000 | 0.0270 | * |
| MVR+TVP | 8.4490 | 2.0940 | 57.6300 | 0.0082 | ** |
| MVP | 6.4240 | 1.2070 | 49.3200 | 0.0394 | * |
| CABG+MVR | 5.8300 | 1.1600 | 42.9100 | 0.0438 | * |
| DVR+TVP | 1.2110 | 0.0527 | 14.1200 | 0.8812 | ns |
| ASD closure | 6.3820 | 0.2769 | 74.7900 | 0.1492 | ns |
| Other | 4.6220 | 1.2550 | 30.1300 | 0.0474 | * |
| **Ablation** |  |  |  |  |  |
| No | Ref. | | | | |
| Yes | 0.8342 | 0.4294 | 1.5890 | 0.5857 | ns |
| **Pump** |  |  |  |  |  |
| Occlusive | Ref. | | | | |
| Centrifugal | 0.6691 | 0.0347 | 3.8990 | 0.7139 | ns |
| **Cardioplegia type** |  |  |  |  |  |
| Crystal | Ref. | | | | |
| Cold blood | 1.1930 | 0.4216 | 3.6620 | 0.7467 | ns |
| **Cardioplegia volume (per ml)** | 1.0000 | 0.9999 | 1.0010 | 0.1610 | ns |
| **Hypothermia** |  |  |  |  |  |
| Mild | Ref. | | | | |
| Moderate | 0.7621 | 0.3954 | 1.3750 | 0.3899 | ns |
| Deep | 0.5279 | 0.0235 | 9.6640 | 0.7280 | ns |
| **Circulation** |  |  |  |  |  |
| Normal | Ref. | | | | |
| Arrested or low-flow | 0.7543 | 0.0358 | 14.4900 | 0.8779 | ns |
| **CPB time (per min)** | 1.0190 | 1.0090 | 1.0280 | 0.0001 | *** |
| **Aortic clamping time (per min)** | 0.9837 | 0.9709 | 0.9969 | 0.0147 | * |

#. Abbreviation: ASD, atrial septal defect; AVR, aortic valve replacement; BMI, body mass index; CABG, coronary artery bypass grafting; CI, confidence interval; CPB, cardiopulmonary bypass; DVR, double valve replacement; MVP, mitral valvuloplasty; MVR, mitral valve replacement; ns, no significance; OR, odds ratio; TVP, tricuspid valvuloplasty.
