# Supplementary material for: Risk assessment of temporary pacing for cardiac arrest after cardiopulmonary bypass-assisted cardiovascular surgery: A case-control study
Source: PLoS One. 2025 May 19;20(5):e0323795. doi: 10.1371/journal.pone.0323795 (PMC12088002; doi:10.1371/journal.pone.0323795)
Supplement: S6 Table — (DOCX) [file pone.0323795.s006.docx]

**S6 Table. The multiple logistic regression without outliers identified by the iterative Grubbs’ method.^#^**

| **Characteristic** | **OR** | **95%CI lower limit** | **95%CI upper limit** | **P value** | **Significance** |
| --- | --- | --- | --- | --- | --- |
| **Sex** |  |  |  |  |  |
| Male | Ref. | | | | |
| Female | 1.1580 | 0.7620 | 1.7630 | 0.4930 | ns |
| **Age (per year)** | 1.0390 | 1.0210 | 1.0570 | <0.0001 | **** |
| **BMI (per kg·m^-2^)** | 0.9967 | 0.9363 | 1.0600 | 0.9167 | ns |
| **Preoperative rhythm** |  |  |  |  |  |
| Sinus rhythm | Ref. | | | | |
| Atrial fibrillation | 3.5830 | 2.1080 | 6.0200 | <0.0001 | **** |
| **Operation** |  |  |  |  |  |
| CABG | Ref. | | | | |
| MVR | 5.6330 | 1.4770 | 37.1200 | 0.0272 | * |
| AVR | 4.2560 | 0.9759 | 29.4800 | 0.0793 | ns |
| DVR | 5.6190 | 1.3990 | 37.9000 | 0.0312 | * |
| MVR+TVP | 8.1400 | 2.1210 | 53.8200 | 0.0076 | ** |
| MVP | 5.0440 | 0.9862 | 37.4300 | 0.0664 | ns |
| CABG+MVR | 6.1410 | 1.3330 | 43.4300 | 0.0313 | * |
| DVR+TVP | 2.6980 | 0.4076 | 22.0600 | 0.3027 | ns |
| ASD closure | 6.2520 | 0.2755 | 71.2800 | 0.1494 | ns |
| Other | 4.2310 | 1.2030 | 26.8600 | 0.0553 | ns |
| **Ablation** |  |  |  |  |  |
| No | Ref. | | | | |
| Yes | 0.9293 | 0.5046 | 1.6920 | 0.8117 | ns |
| **Pump** |  |  |  |  |  |
| Occlusive | Ref. | | | | |
| Centrifugal | 1.2010 | 0.1779 | 4.7560 | 0.8192 | ns |
| **Cardioplegia type** |  |  |  |  |  |
| Crystal | Ref. | | | | |
| Cold blood | 0.9594 | 0.3751 | 2.7230 | 0.9340 | ns |
| **Cardioplegia volume (per ml)** | 1.0000 | 0.9998 | 1.0000 | 0.5507 | ns |
| **Hypothermia** |  |  |  |  |  |
| Mild | Ref. | | | | |
| Moderate | 0.7574 | 0.4136 | 1.3160 | 0.3443 | ns |
| Deep | 0.3571 | 0.0276 | 3.8180 | 0.4594 | ns |
| **Circulation** |  |  |  |  |  |
| Normal | Ref. | | | | |
| Arrested or low-flow | 1.5070 | 0.1200 | 16.6500 | 0.7683 | ns |
| **CPB time (per min)** | 1.0100 | 1.0010 | 1.0170 | 0.0129 | * |
| **Aortic clamping time (per min)** | 0.9938 | 0.9828 | 1.0050 | 0.2790 | ns |

#. Abbreviation: ASD, atrial septal defect; AVR, aortic valve replacement; BMI, body mass index; CABG, coronary artery bypass grafting; CI, confidence interval; CPB, cardiopulmonary bypass; DVR, double valve replacement; MVP, mitral valvuloplasty; MVR, mitral valve replacement; ns, no significance; OR, odds ratio; TVP, tricuspid valvuloplasty.
