# Supplementary material for: Risk assessment of temporary pacing for cardiac arrest after cardiopulmonary bypass-assisted cardiovascular surgery: A case-control study
Source: PLoS One. 2025 May 19;20(5):e0323795. doi: 10.1371/journal.pone.0323795 (PMC12088002; doi:10.1371/journal.pone.0323795)
Supplement: S7 Table — (DOCX) [file pone.0323795.s007.docx]

**S7 Table. The multiple logistic regression with binning continuous variables.^#^**

| **Characteristic** | **OR** | **95%CI lower limit** | **95%CI upper limit** | **P value** | **Significance** |
| --- | --- | --- | --- | --- | --- |
| **Sex** |  |  |  |  |  |
| Male | Ref. | | | | |
| Female | 1.1690 | 0.7714 | 1.7750 | 0.4616 | ns |
| **Age group** |  |  |  |  |  |
| Young | Ref. | | | | |
| Old | 2.0210 | 1.3270 | 3.0590 | 0.0009 | *** |
| **BMI (per kg·m^-2^)** |  |  |  |  |  |
| Normal | Ref. | | | | |
| Underweight | 1.0080 | 0.5154 | 1.8510 | 0.9816 | ns |
| Overweight | 1.1140 | 0.6695 | 1.8110 | 0.6687 | ns |
| Obesity | 1.4430 | 0.6351 | 2.9580 | 0.3448 | ns |
| **Preoperative rhythm** |  |  |  |  |  |
| Sinus rhythm | Ref. | | | | |
| Atrial fibrillation | 0.2611 | 0.1559 | 0.4425 | <0.0001 | **** |
| **Operation** |  |  |  |  |  |
| CABG | Ref. | | | | |
| MVR | 6.2110 | 1.6220 | 41.0400 | 0.0199 | * |
| AVR | 4.1930 | 0.9612 | 29.0400 | 0.0824 | ns |
| DVR | 5.3210 | 1.3410 | 35.7000 | 0.0360 | * |
| MVR+TVP | 8.6960 | 2.2400 | 57.8700 | 0.0062 | ** |
| MVP | 4.8490 | 0.9559 | 35.8300 | 0.0722 | ns |
| CABG+MVR | 5.6660 | 1.2280 | 40.0900 | 0.0398 | * |
| DVR+TVP | 2.4580 | 0.3706 | 20.1200 | 0.3508 | ns |
| ASD closure | 4.6580 | 0.2062 | 52.6600 | 0.2247 | ns |
| Other | 4.1200 | 1.1700 | 26.1900 | 0.0602 | ns |
| **Ablation** |  |  |  |  |  |
| No | Ref. | | | | |
| Yes | 0.8510 | 0.4629 | 1.5480 | 0.5994 | ns |
| **Pump** |  |  |  |  |  |
| Occlusive | Ref. | | | | |
| Centrifugal | 0.9960 | 0.1470 | 3.9360 | 0.9960 | ns |
| **Cardioplegia type** |  |  |  |  |  |
| Crystal | Ref. | | | | |
| Cold blood | 1.0350 | 0.4252 | 2.8330 | 0.9423 | ns |
| **Cardioplegia volume (per ml)** |  |  |  |  |  |
| Small | Ref. | | | | |
| Medium | 1.3390 | 0.7202 | 2.5650 | 0.3659 | ns |
| Large | 1.3480 | 0.6388 | 2.8740 | 0.4352 | ns |
| **Hypothermia** |  |  |  |  |  |
| Mild | Ref. | | | | |
| Moderate | 1.6360 | 0.1691 | 8.6070 | 0.6201 | ns |
| Deep | 0.7560 | 0.4223 | 1.2880 | 0.3230 | ns |
| **Circulation** |  |  |  |  |  |
| Normal | Ref. | | | | |
| Arrested or low-flow | 0.3617 | 0.0531 | 3.5720 | 0.3417 | ns |
| **CPB time (per min)** |  |  |  |  |  |
| Short | Ref. | | | | |
| Medium | 1.2250 | 0.5864 | 2.5110 | 0.5828 | ns |
| Long | 2.5020 | 1.0540 | 5.8220 | 0.0351 | * |
| **Aortic clamping time (per min)** |  |  |  |  |  |
| Short | Ref. | | | | |
| Medium | 1.1150 | 0.5744 | 2.2460 | 0.7534 | ns |
| Long | 0.7603 | 0.3192 | 1.8700 | 0.5425 | ns |

#. Abbreviation: ASD, atrial septal defect; AVR, aortic valve replacement; BMI, body mass index; CABG, coronary artery bypass grafting; CI, confidence interval; CPB, cardiopulmonary bypass; DVR, double valve replacement; MVP, mitral valvuloplasty; MVR, mitral valve replacement; ns, no significance; OR, odds ratio; TVP, tricuspid valvuloplasty.
