# Supplementary material for: Risk assessment of temporary pacing for cardiac arrest after cardiopulmonary bypass-assisted cardiovascular surgery: A case-control study
Source: PLoS One. 2025 May 19;20(5):e0323795. doi: 10.1371/journal.pone.0323795 (PMC12088002; doi:10.1371/journal.pone.0323795)
Supplement: S8 Table — (DOCX) [file pone.0323795.s008.docx]

**S8 Table. The longest CPB time with limited temporary pacing risk in different ages.***

| **Age (years)** | **CPB time (min)** |
| --- | --- |
| 0 | 350 |
| 1 | 341.25 |
| 2 | 332.5 |
| 3 | 323.75 |
| 4 | 315 |
| 5 | 306.25 |
| 6 | 297.5 |
| 7 | 288.75 |
| 8 | 280 |
| 9 | 271.25 |
| 10 | 262.5 |
| 11 | 253.75 |
| 12 | 245 |
| 13 | 236.25 |
| 14 | 227.5 |
| 15 | 218.75 |
| 16 | 210 |
| 17 | 201.25 |
| 18 | 192.5 |
| 19 | 183.75 |
| 20 | 175 |
| 21 | 166.25 |
| 22 | 157.5 |
| 23 | 148.75 |
| 24 | 140 |
| 25 | 131.25 |
| 26 | 122.5 |
| 27 | 113.75 |
| 28 | 105 |
| 29 | 96.25 |
| 30 | 87.5 |
| 31 | 78.75 |
| 32 | 70 |
| 33 | 61.25 |
| 34 | 52.5 |
| 35 | 43.75 |
| 36 | 35 |
| 37 | 26.25 |
| 38 | 17.5 |
| 39 | 8.75 |

*. The results are determined by the scoring system, score=*age (year)*/40+*CPB time (min)*/350+[*preoperative atrial fibrillation*]×1. Abbreviation: CPB, cardiopulmonary bypass.
