# Supplementary material for: Risk assessment of temporary pacing for cardiac arrest after cardiopulmonary bypass-assisted cardiovascular surgery: A case-control study
Source: PLoS One. 2025 May 19;20(5):e0323795. doi: 10.1371/journal.pone.0323795 (PMC12088002; doi:10.1371/journal.pone.0323795)
Supplement: Attachment — Submitted filename: Comments for 58174.docx [file pone.0323795.s009.docx]

**Review of the Manuscript: Risk Assessment of Temporary Pacing for Cardiac Arrest After Cardiopulmonary Bypass-Assisted Cardiovascular Surgery: A Case-Control Study**

The manuscript presents a case-control study on the risk assessment of temporary pacing for cardiac arrest following cardiopulmonary bypass (CPB)-assisted cardiovascular surgery. The study addresses an important clinical question, offering a scoring system to evaluate risk factors. However, there are areas that require revision, including the clarity of the methodology, statistical justification, and overall presentation of results. Below are specific comments regarding strengths and areas for improvement.

**Strengths:**

1. **Clinical Relevance:** The study addresses a significant clinical concern, providing a risk assessment for temporary pacing after cardiac surgery.
2. **Large Sample Size:** The study includes 2,326 patients, which strengthens the statistical power.
3. **Clear Identification of Risk Factors:** Independent risk factors such as age, preoperative atrial fibrillation, and prolonged CPB time are well-identified and explained.
4. **Development of a Scoring System:** The creation of a risk stratification model adds practical value for clinical decision-making.

**Major Concerns:**

**1. Abstract Clarity and Precision**

- The abstract should explicitly state the statistical methods used to determine independent risk factors and scoring system development.
- Clarify the meaning of “certain indications” in the methods section.
- The conclusion should emphasize the clinical applicability of the scoring system.

**2. Methodology and Statistical Analysis**

- The description of how patients were categorized into case and control groups lacks clarity. The inclusion and exclusion criteria need refinement, particularly regarding preoperative ventricular arrhythmia.
- The statistical justification for using specific cut-offs in logistic regression models should be provided.
- The rationale for choosing coronary artery bypass grafting (CABG) as the reference category in operation type comparisons should be explained.
- Multiple logistic regression: The criteria for stepwise elimination should be described in greater detail.
- The scoring system parameters need validation against an external dataset. If not feasible, a discussion on its limitations should be included.

**3. Presentation of Results**

- Tables 1 and 2 should be reformatted for clarity. Consider summarizing key findings in the text.
- Figure 2D requires additional explanation to clarify how likelihood ratios were determined.
- Sensitivity and specificity should be interpreted in a clinical context—what constitutes an acceptable threshold?

**4. Discussion and Clinical Implications**

- The study should compare findings with existing literature on temporary pacing after CPB.
- Potential confounding factors such as perioperative medications, patient comorbidities, and intraoperative hemodynamics should be acknowledged.
- The limitations section should expand on how surgical techniques and institutional practices may affect generalizability.

**5. Language and Formatting**

- Several grammatical errors and awkward phrasings are present. A professional language review is recommended.
- Consistency in terminology: The manuscript alternates between “temporary pacing” and “temporary pacemaker use” without clear distinction.
- Reference formatting should follow journal guidelines.

**Minor Comments:**

1. Define abbreviations upon first use.
2. Ensure figures are high resolution and appropriately labeled.
3. The ethical approval statement should be moved to the methods section.

This study presents valuable findings on temporary pacing after CPB-assisted cardiovascular surgery. However, revisions are necessary to enhance methodological clarity, statistical justification, and the clinical interpretation of results. Addressing these concerns will significantly strengthen the manuscript's impact and readability.
