# Supplementary material for: Risk assessment of temporary pacing for cardiac arrest after cardiopulmonary bypass-assisted cardiovascular surgery: A case-control study
Source: PLoS One. 2025 May 19;20(5):e0323795. doi: 10.1371/journal.pone.0323795 (PMC12088002; doi:10.1371/journal.pone.0323795)
Supplement: Attachment — Submitted filename: Response to Reviewers.docx [file pone.0323795.s012.docx]

**Responses to the reviewers**

(1) Reviewer #1

Q1: The abstract does not provide sufficient background to contextualize the importance of studying temporary pacing. Suggestion: Add 1-2 sentences explaining the clinical implications of temporary pacing and its relevance to postoperative outcomes.

A1: We added the background that explains why we need to carry out this study in the Abstract-Objective part. The clinical context is also further introduced in the Discussion 4.1 part. Briefly, the key problem is that (1) the high risk (49.4%) of mortality caused by postoperative cardiac arrest should be timely rescued by temporary pacing although the incidence was low (0.7%-5.2%), but (2) routine pacing wire insertion can cause noteworthy (1.74%) iatrogenic complications, considering the large number of patients that do not develop a cardiac arrest. Our work aimed to identify as many patients that do not develop a cardiac arrest to reduce the use of pacing wires.

Q2: The results section is data-heavy, with numerous ORs and CIs presented in a dense format that might overwhelm readers.

A2: We adjusted data presenting in Table 1: (1) P values were mostly rounded to 3 decimal places; (2) original data were rounded to up to 2 decimal place. We also replaced the Table 2 with forest plots in Fig 2, which makes the results easy to be read.

Q3: While the abstract mentions the scoring system, it does not explain how the scoring system was developed or validated.

A3: We wrote separated sections, Method 2.5 and 2.7, to introduce how the scoring system was developed. The scoring system was validated using the same cases and its performance of excluding patients that do not develop cardiac arrest achieved 0% false negative rate, which meets the clinical need.

Q4: The conclusion reiterates findings but does not address the scoring system’s potential for clinical implementation or its limitations.

A4: We rewrote the conclusion in Abstract and the main text. The clinical implementation was clarified as the scoring system was established to exclude the patients with low risks of cardiac arrest. The limitations were placed in the Discussion part 4.4.

Q5: Terms like "long CPB time" are vague and lack specific thresholds, which might limit reproducibility.

A5: To make our results clear, we provided the S8 Table. It guides readers to find the longest CPB time that does not significantly increase the risk of cardiac arrest in different ages.

Q6: The abstract does not acknowledge any study limitations, which could affect the interpretation of findings.

A6: We agree with your opinion that the lack of limitations in Abstract may cause a misinterpretation of results, while with all necessary information presented, we reached the 300-word limit of Abstract. We ensured that the limitations are fully discussed in the Discussion 4.4 part.

Q7: Replace "Odd ratio" with "Odds ratio" for accuracy.

A7: Thank you for pointing out this spelling mistake. It has been corrected in the text and figures.

Q8: Scoring System AUC. While the ROC curve AUC of 0.7402 is acceptable, its clinical significance is not addressed. How well does the scoring system perform in different patient populations?

A8: As is guided by the STROBE guidance, we added subgroup analyses in the Result 4.4 part to analyze the system’s performances in different patient populations.

Q9: Preoperative Atrial Fibrillation: This is identified as a major risk factor. The abstract could mention its prevalence in the study cohort for context.

A9: The prevalence of atrial fibrillation was 396 (17.02% in all cases, and 50 in paced cases). It is necessary to write the prevalence of characteristic when it has limited absolute number, while here the atrial fibrillation prevalence was not small (low) in both case and control groups, and we believe that the data did not cause a misinterpretation. Considering limited words in Abstract, please understand that we cannot add the data there.

(2) Reviewer #2

Q1: This study provides clear exclusion criteria, there is a potential risk of collection bias, given that the data was collected by specific team members during working hours. The authors should clarify if there are inherent biases like selection bias, recall bias, and missing data issues, which could have affected the comprehensiveness of their findings.

A1: We agree that there are biases in our study. Since the data collection details are required according to the STROBE guidance, we wrote the members and details of data collection, and ensured that the standard data collection strategies planned by the supporting projects were obeyed, which well minimized the selection bias. In our opinion, the most “solid” bias may originate from the retrospective nature of our study and we fully acknowledged the possible biases, including the ones you mentioned here, in the limitation part.

Q2: While the authors report an AUC of 0.7400 for their scoring system, external validation is critical. In this case, where no external validation exists, caution must be emphasized, as validation in an independent cohort would test the robustness and generalizability of the scoring system. For example, the clinical effectiveness of this model needs to be evaluated prospectively in other hospitals or surgical settings. Furthermore, while the scoring system’s sensitivity (59.26%) and specificity (80.16%) are reported, it would be helpful to provide additional details on the performance of the model at different score cutoffs. How does the model perform in different patient subgroups (e.g., those with comorbidities, and specific valve surgeries)?

A2: We agree that an external set of cohort for validation is crucial, especially in some machine learning models. The difficulty is that our study topic was rarely studied currently and we cannot find a similar research with individual data. An alternative way is to separate a group of cases as the external set, however, the major problem is that the positive (cases who were paced) sample size is small (108 cases) as some subgroups only have less than 5 positive cases, which can cause significant selection bias and be questioned by other researchers. Therefore, we chose to test our scoring system in the same cases, which has been commonly used in medical studies.

In our study, we investigated 2 cutoffs. The first cutoff, as is mentioned here (Sen 59.26%, Spe 80.16%), was determined by the Youden’s index, and this method approaches the balanced, largest Sen and Spe values, however, it is not suitable in the clinical use, because if a patient who finally develops a cardiac arrest but he or she did not have a wire inserted, there can be severe events. The second cutoff was determined by the negative likelihood ratio (please see Method 2.7 and Result 3.4), and this cutoff identifies the patients who can be excluded from wire insertion, which can wrongly identify patients who are cardiac arrest-negative as cardiac arrest-positive but never identify those who are positive as negative. The second cutoff is more helpful here, because it helps patients and surgeons to efficiently avoid a part of unnecessary wire insertion.

We did not have enough comorbidity data for analysis, but we performed subgroup analyses in different subgroups of procedures. We agree that the subgroup analyses are necessary, because having the valve surgery was identified as a risk factor but it was not included in the scoring system.

Q3: The authors highlight that non-CABG operations (e.g., MVR, DVR) are independent risk factors for temporary pacing. However, the paper does not discuss how the complexity or specific technical aspects of these procedures may increase the likelihood of pacing. For instance, does mitral valve replacement inherently cause more myocardial stress or post-operative arrhythmias? Further discussion could provide a more mechanistic understanding of these findings.

A3: Thanks for your reminding. We did not deeply think about the reason why non-CABG operations can increase the risk of temporary pacing. Here we inserted the reference [21] and briefly introduced the most acceptable explanation currently available, i.e. the valvular surgery procedures can cause injuries in atrioventricular conduction axis because the replacing/repairing area is close to this axis. Additionally, we also discussed other risk factors in the Discussion part.

Q4: The study focuses on the need for temporary pacing, including other postoperative outcomes such as mortality, long-term arrhythmias, and recovery times. These additional outcomes would provide a more comprehensive assessment of the clinical implications of temporary pacing. The authors didn’t mention if temporary pacing leads to a need for permanent pacing in some patients. Including this information would be valuable, as it would indicate whether the need for temporary pacing might signal a higher risk for long-term pacing requirements.

A4: Due to limited literature we found and data we have, we cannot draw a conclusion that permanent pacing is positively correlated with temporary pacing, but studies on single temporary or permanent pacing have found common risk factors. Here we inserted a report ([27]) published on BJCVS, and it found that the in hospital mortality was 10% higher in temporary paced patients compared with those who were not paced. We put it into the Limitations part and admit that this is a shortage of our study.

(3) Reviewer #3

Q1: The retrospective design is appropriate given the clinical context; however, additional clarification on the selection criteria (and potential selection biases) would strengthen the manuscript. The exclusion of patients with incomplete information or preoperative pacing is clearly justified, but the manuscript should discuss how these exclusions might affect generalizability.

A1: In the Limitation part, we wrote that selection bias can influence the results through personal factors. As for the exclusion criteria, your advice remind us of a key problem that our recording system was once updated in 2018, which may influence the data recording process and this potential bias was also added to the Limitation part. Some patients were excluded for preoperative ventricular arrhythmia and artificial pacing, while this proportion of patients is quite small and the patients’ basic conditions have a definite influence on the outcome, thus we believe that their exclusion does not affect the model’s generalizability.

Q2: The logistic regression analysis appears robust, and the identification of independent risk factors is supported by appropriate statistical tests. The development of the scoring system is innovative; however, external validation in an independent cohort is necessary to confirm its clinical utility. The manuscript would benefit from a more detailed discussion of the model’s calibration (beyond the Hosmer-Lemeshow test) and discrimination metrics.

A2: Since Prime 9.5 only provides limited tools, we added 2 additional parts for the model’s calibration according to the STROBE guideline. In Method part 2.4+Result part 3.2, we performed the sensitive analyses to assess the robustness of the independent risk factors we found. In Method part 2.6+Result 3.3, we applied the scoring system to different operation type subgroups to assess the system’s generalizability in different patients.

Q3: The authors effectively argue that temporary pacing is an important intervention with non-negligible risks, and that predicting its necessity could optimize patient management. It would be useful to elaborate on how the scoring system might be integrated into clinical practice and whether it might reduce unnecessary temporary pacing wire insertions.

A3: In the Discussion 4.1 part, we wrote the problem of current clinical strategy: (1) the high risk (49.4%) of mortality caused by postoperative cardiac arrest should be timely rescued by temporary pacing although the incidence was low (0.7%-5.2%), but (2) routine pacing wire insertion can cause noteworthy (1.74%) iatrogenic complications, considering the large number of patients that do not develop a cardiac arrest. In the Discussion 4.2 part, we wrote the reason why the quantitative scoring system along with the cutoff determined by negative likelihood ratio is useful and better than some “traditional” binary outcome prediction models, as we only exclude patients with a very low risk of cardiac arrest. This strategy is quite practical in clinical decisions.

Q4: Some sections of the Methods and Results could be more concise; consider streamlining the text to focus on the most critical details.

A4: In this revision, we separated multiple logistic regression, sensitivity analysis, scoring system establishing, subgroup analysis, likelihood ratio calculation into different parts. The results include 4 parts: general information of the patients, risk factor verification, the scoring system’s performance, and its use in the clinical context with a cutoff determined by negative likelihood ratio. This organization can better illustrate the contents.

Q5: The description of the risk factors (e.g., CPB time, preoperative rhythm) is clear, but providing a table that summarizes baseline characteristics between the pacing and non-pacing groups would enhance clarity.

A5: Please find the baseline information in Table.1. Recently, we read that some researchers advised not comparing demographics information in Table.1, but we still made statistical analyses comparing the non-pacing and pacing groups. We did not draw conclusions from this table since there can be a Simpson's paradox in non-RCT cohorts.

Q6: Figures and supplementary materials (such as the ROC curve and distribution of scores) are helpful; ensure they are of high resolution and fully referenced in the text. There are a few typographical errors and minor formatting issues that should be corrected prior to publication.

A6: Thanks for your advice. Before this submission, we checked the fig quality using the tool provided by the journal. We carefully checked the spelling and grammar in the revised manuscript.

(4) Reviewer #4

Q1: Abstract Clarity and Precision

• The abstract should explicitly state the statistical methods used to determine independent risk factors and scoring system development.

• Clarify the meaning of “certain indications” in the methods section.

• The conclusion should emphasize the clinical applicability of the scoring system.

A1: We reorganized the Abstract. The methods of statistical analyses (specifically, multiple logistic regression), scoring system establishing and the study’s clinical applicability was added to the Method part and the Conclusion part, respectively. We wrote a background in the Objective part that clarifies the indications (asystole or severe bradycardia) of pacing.

Q2: Methodology and Statistical Analysis

• The description of how patients were categorized into case and control groups lacks clarity. The inclusion and exclusion criteria need refinement, particularly regarding preoperative ventricular arrhythmia.

A2-1: We verily recorded these processes, and although they are simpler than prospective studies, no additional criterion or standard was applied. Since the study design is case-control, the categorizing process may be mostly influenced by the information bias; the inclusion and exclusion processes may be influenced by the selection bias, and we added these possible problems in the Limitation part. The 2 preoperative ventricular arrhythmia cases were ventricular tachycardia and degree III atrioventricular block, respectively, and we added this information in the Result 4.1 part.

• The statistical justification for using specific cut-offs in logistic regression models should be provided.

A2-2: We described the Youden’s index calculation that determined the cutoff (2.282) with Sen 59.26%, Spe 80.16% in the Method 2.5 part. Another cutoff (≤1) was determined by the negative likelihood ratio that can exclude the need of temporary pacing, which is described in Method 2.7 part.

• The rationale for choosing coronary artery bypass grafting (CABG) as the reference category in operation type comparisons should be explained.

A2-3: We added the reason “for its external, non-valvular feature of procedure that less affects the conduction system.” in Method 2.3 part. The CABG procedure does not open the heart and has operations on the heart surface, which less affects the conduction system deeply organized in the heart and has been believed to have lower risks of pacing. Further texts explaining the anatomical mechanisms of valvular surgery were added in the Discussion 4.3 part.

• Multiple logistic regression: The criteria for stepwise elimination should be described in greater detail.

A2-4: We added these details in the Method part 2.5. The method is well established and other scoring systems such as the H_2_FPEF score for heart failure of preserved ejection fraction also used this method.

• The scoring system parameters need validation against an external dataset. If not feasible, a discussion on its limitations should be included.

A2-5: Due to limited studies on this topic, we cannot obtain external data for the validation. An alternative way is to separate a group of cases as the external set, however, the major problem is that the positive (cases who were paced) sample size is small (108 cases), and some subgroups only have less than 5 positive cases, which can cause significant selection bias and be questioned by other researchers. Therefore, we chose to test our scoring system in the same cases, which has been commonly used in medical studies. To have the necessary validation, we performed sensitivity analysis (Result 3.2) to test the robustness of risk factors’ contribution to pacing, and subgroup analysis (Result 3.3) to test the generalizability of the risk scores in different operation types, according to the STROBE guidance. Considering a high false positive rate, we also added a limitation point in the Limitation part.

Q3: Presentation of Results

• Tables 1 and 2 should be reformatted for clarity. Consider summarizing key findings in the text.

A3-1: We adjusted data presenting in Table 1: (1) P values were rounded to 3 decimal places, (2) original data were rounded to up to 2 decimal place. We also replaced the Table 2 with forest plots in Fig 2, which makes the results easier to be read.

• Figure 2D requires additional explanation to clarify how likelihood ratios were determined.

A3-2: The calculation of likelihood ratio has been written in Method 2.7 part, and we provided a citation [20] that explains how the index works in clinical applications in detail.

• Sensitivity and specificity should be interpreted in a clinical context—what constitutes an acceptable threshold?

A3-3: The key problem is that (1) the high risk (49.4%) of mortality caused by postoperative cardiac arrest should be timely rescued by temporary pacing although the incidence was low (0.7%-5.2%), but (2) routine pacing wire insertion can cause noteworthy (1.74%) iatrogenic complications, considering the large number of patients that do not develop a cardiac arrest. Our work aimed to identify as many patients that do not develop a cardiac arrest to reduce the use of pacing wires. And here the threshold determination of scoring system is discussed in the Discussion 4.2 part.

Q4: Discussion and Clinical Implications

• The study should compare findings with existing literature on temporary pacing after CPB.

A4-1: The study of our topic, though we believe that it is important, has rarely been investigated in existing literature. The key blank is that there is no quantitative risk assessment method for case exclusion in the up-mentioned clinical context, and we only find some articles discussing low-risk procedures such as pediatric congenital heart disease operations and CABG. We added these articles in the Discussion 4.1 part and also pointed out their shortages in the Discussion 4.2 part.

• Potential confounding factors such as perioperative medications, patient comorbidities, and intraoperative hemodynamics should be acknowledged.

A4-2: Thanks for your reminding of these confounding factors. We added them in the Limitation part.

• The limitations section should expand on how surgical techniques and institutional practices may affect generalizability.

A4-3: We added the reason “as the clinical outcomes of patients undergoing cardiovascular surgery vary significantly due to the complexity of practices, including in-hospital management, anesthesia, and CPB” to this limitation sentence, which explains how surgical techniques and institutional practices may affect generalizability.

Q5: Language and Formatting

• Several grammatical errors and awkward phrasings are present. A professional language review is recommended.

• Consistency in terminology: The manuscript alternates between “temporary pacing” and “temporary pacemaker use” without clear distinction.

• Reference formatting should follow journal guidelines.

• Define abbreviations upon first use.

• Ensure figures are high resolution and appropriately labeled.

• The ethical approval statement should be moved to the methods section.

A5: Thanks for your advice. We carefully checked the language problem before this submission. The terms like “temporary pacemaker use” are unified. Figures were checked by the tools provided by the journal. Other adjustments were made accordingly as well.

(5) Reviewer #5

Q1: Abstract

Spell out the numbers two thousand three hundred twenty-six and one hundred eight as they appear at the start of the sentence.

Rephrase: A scoring formula was developed, achieving an area under the receiver operating characteristic (ROC) curve (AUC) of 0.7402 (95% CI: 0.6939–0.7865, P < 0.0001).

Revise: A scoring system incorporating age, preoperative rhythm, and CPB time can quantitatively assess the associated risk.

A1: The sentence was rewrote as “The scoring system, score=age (year)/40+CPB time (min)/350+[preoperative atrial fibrillation]×1, can quantitatively assess the associated risk with an area under receiver of characteristic (ROC) curve (AUC) of 0.74 (95% confidential interval 0.69-0.79) (P<0.001)”. We believe that the formula and AUC are important results that readers would like to find in the Abstract, so we left them in the sentence. Please understand that we reached the word limit of Abstract and spelling out the number can take a large space.

Q2: Methods

Since this is a case-control study, explicitly state the inclusion and exclusion criteria for both cases and controls.

Justify why this study is not classified as a retrospective cohort study.

A2-1: I’m (Wang, H) pleased that we have a chance to discuss this interesting question.

We noticed that currently there are many comments on existing literature that criticizing the definition of study design in the title and text, and the confusion of retrospective cohort study and case-control study is commonly seen. Upon definition, the key difference between these study designs is the causation inferring process, i.e. the retrospective cohort study defines two groups of patients with different exposures and checks their outcomes after a certain period, while the case-control study collects two groups of patients with different outcomes and checks their exposures. Among the many problems the confusion may bring, the worst one is missing the fact that case-control study can not calculate the incidence (or report a relative risk) because the case or control outcome selection results in a non-natural outcome “abundance”.

According to our study design, we grouped the patients according to the outcomes we investigated, namely whether having temporary pacing, and compared the exposures between groups, which meets the definition of case-control study. With the same cases we have, it is also reasonable to perform a retrospective cohort study, and if so, we may choose several exposures to define the control group and exposed group, and check their outcomes after surgical procedures. Then we can have 4 outcomes, namely paced cases and not paced cases in each of the 2 initial groups. However, we did not choose a retrospective cohort study, because we aimed to develop a scoring system for the very outcomes we focused on, and a case-control design is very suitable for the imbalanced outcomes with only 4.64% positive cases.

In this study, we had the same inclusion and exclusion criteria in both groups, which is a little bit different from some other case-control studies. In certain studies carried out by epidemiologists, the exposures of a certain disease, for instance, in a city, are rather complicated, and if they want to compare the exposures between the diseased cases and non-diseased controls, it is very necessary to make a plan for control inclusion, as it is almost impossible to compare the cases with all non-diseased citizens. Somewhat differently, here in our study, the patients included underwent similar CPB-assisted procedures, and therefore, we can simply analyze all patients and divide them into case and control groups only depending on the outcomes. This feature also allowed us to calculate the incidence of cardiac arrest.

Revise the statistical analysis section: Specify where the D’Agostino & Pearson test, Mann-Whitney test, chi-square test or Fisher’s exact test, and Baptista-Pike method were applied in the results.

A2-2: We specified that these statistical analyses were performed in Table 1 (demographics and operational information).

Q3: Results

Spell out the numbers two thousand four hundred forty-six and one hundred nine as they appear at the start of the sentence.

Report p-values to three decimal places, percentages to one decimal place, and other values to two decimal places.

A3-1: We changed the data presentation in the main text according to your advice. In the scoring system table, we left the original decimal places because these values were used to illustrate how the system was developed. We appreciate your confirmation of our sample size, but please understand that we believe that the arabic number can present these results better, so the original numbers were left unchanged.

Table 1:

-This table should report results from simple logistic regression.

-Replace "statistics" with p-values.

-Reclassify some variables where necessary.

-Ensure the reference category is reported first (e.g., "male" followed by "female").

A3-2: We added simple logistic regression results (95%CI) to Table 1. The word “statistics” was replaced. Classifications of variables were done in a sensitivity analysis (S8 Table), but we did not use the categorized variables in the scoring system because they cannot achieve the goal of excluding low-risk cases.

Table 3: Clearly state the variable selection method used.

A3-3: The variable selection method is placed in the Method 2.5 part.

Revise:

-The incidence of temporary pacing increased with the score. When the score was below 2, the incidence remained below 5% (Fig. 2C).

-With an interval of 0.002, cutoff values ≤1.138 or ≥3.474 corresponded to negative likelihood ratios <0.1 and positive likelihood ratios >10 (Fig. 2D).

A3-4: Thank you for providing these sentences with good expression.

Q4: Discussion and Reference

Expand this section as it is currently too brief.

Revise the use of the term "incidence" to ensure accuracy.

Discuss why the type of operation was not included in the scoring system.

Address the exclusion of several key factors and how this impacts the scoring system.

Additional references should be included where relevant.

A4: The discussion part and literature citations were extended in this submission. We provided the reason why we did not include the operation type in the scoring system, “Firstly, the composition of operation types often varies across different centers; secondly, it is highly susceptible to the influence of the surgeon's skill; thirdly, incorporating this factor would increase the complexity of the scoring system, potentially hindering its clinical applicability”. Additionally, we did subgroup analyses in different operation types. As for the exclusion of potential key factors, we can see that the AUC of the scoring system (0.74) was not significantly decreased compared with the initial MLR result (0.76), which can validate that our stepwise elimination method performed well in feature selection. According to the epidemiology terminology, please understand that we believe the word “incidence” is proper to describe the proportion of patients who have some events in a cohort.
