# Supplementary material for: Risk assessment of temporary pacing for cardiac arrest after cardiopulmonary bypass-assisted cardiovascular surgery: A case-control study
Source: PLoS One. 2025 May 19;20(5):e0323795. doi: 10.1371/journal.pone.0323795 (PMC12088002; doi:10.1371/journal.pone.0323795)
Supplement: Attachment — Submitted filename: Comments for 58174 R1.docx [file pone.0323795.s011.docx]

**Critical Review of the Manuscript: Risk Assessment of Temporary Pacing for Cardiac Arrest After Cardiopulmonary Bypass-Assisted Cardiovascular Surgery**

**General Overview**

This study aims to assess the risk of temporary pacing in patients experiencing cardiac arrest after CPB-assisted cardiovascular surgery. The authors employ multiple logistic regression (MLR) to identify independent risk factors and develop a scoring system for clinical application. While the study is methodologically sound and clinically relevant, several areas require refinement to enhance clarity, validity, and applicability.

**Strengths**

1. **Relevance and Novelty**
   - The topic addresses an important clinical dilemma: balancing the benefits of temporary pacing with the risks of unnecessary pacing wire insertion.
   - The study attempts to fill a gap in current literature by providing a quantitative assessment model.
2. **Methodological Rigor**
   - The study includes a large sample size (n=2326), enhancing statistical power.
   - The use of MLR is appropriate for identifying independent risk factors.
   - Sensitivity analysis and subgroup analysis strengthen the robustness of findings.
3. **Development of a Practical Tool**
   - The scoring system offers a structured approach for identifying patients at low risk of requiring pacing, potentially reducing unnecessary interventions.
   - The chosen cutoff (score ≤1) minimizes false negatives, ensuring that high-risk patients are not overlooked.

**Areas for Improvement**

1. **Clarity in Presentation**
   - The manuscript presents results in a highly technical manner. Simplifying the text and using clearer tables or graphical representations (such as summary tables of key findings) would improve readability.
   - The replacement of Table 2 with forest plots is helpful, but further effort is needed to make the statistical findings more accessible to clinicians.
2. **Validation of the Scoring System**
   - The absence of external validation is a significant limitation. While the authors acknowledge this, they should provide a more detailed discussion on the necessity of validating the model in independent cohorts.
   - The current validation approach (testing the scoring system within the same dataset) may introduce bias and overestimate model performance.
3. **Consideration of Confounding Factors**
   - The study does not fully explore perioperative factors (e.g., medication use, intraoperative hemodynamics, and surgeon expertise) that may influence pacing needs.
   - A more detailed discussion on how the type of surgery affects pacing risk is necessary. For example, why do non-CABG procedures increase the likelihood of pacing?
4. **Limitations and Bias**
   - The manuscript acknowledges selection bias but does not sufficiently elaborate on recall bias, missing data issues, or potential inaccuracies in data collection.
   - The retrospective design limits causal inference, which should be explicitly stated in relation to future prospective studies.
5. **Clinical Implementation**
   - The discussion should elaborate on how the scoring system could be integrated into clinical workflows.
   - Exploring the potential for incorporation into electronic health records (EHRs) for automated decision support would enhance practical utility.

**Conclusion**

This study contributes valuable insights into the risk assessment of temporary pacing post-CPB-assisted cardiovascular surgery. While the methodology is sound and the scoring system has potential clinical utility, improvements in clarity, external validation, and discussion of confounding factors would further strengthen its impact. Addressing these aspects would make the research more applicable to a broader cardiovascular surgical audience.
