# Supplementary material for: Risk assessment of temporary pacing for cardiac arrest after cardiopulmonary bypass-assisted cardiovascular surgery: A case-control study
Source: PLoS One. 2025 May 19;20(5):e0323795. doi: 10.1371/journal.pone.0323795 (PMC12088002; doi:10.1371/journal.pone.0323795)
Supplement: Attachment — Submitted filename: Response_to_reviewers_auresp_2.docx [file pone.0323795.s013.docx]

**Responses to the reviewers**

(1) Reviewer #4

**1. Clarity in Presentation**

The manuscript presents results in a highly technical manner. Simplifying the text and using clearer tables or graphical representations (such as summary tables of key findings) would improve readability. The replacement of Table 2 with forest plots is helpful, but further effort is needed to make the statistical findings more accessible to clinicians.

Thanks for your valuable advice over this time. We see that Plos ONE has a broad readership and added the schematics to illustrate the workflow of our study. Please see the new Fig 6 in this version.

**2. Validation of the Scoring System**

The absence of external validation is a significant limitation. While the authors acknowledge this, they should provide a more detailed discussion on the necessity of validating the model in independent cohorts. The current validation approach (testing the scoring system within the same dataset) may introduce bias and overestimate model performance.

To further discuss the validation shortness in our study, we added these sentences in the limitation part “This is a single-center study, and the conclusions should be validated using external datasets. To avoid selection bias from the small positive case sample size, we did not separate a validation group. However, testing the scoring system within the same dataset may overestimate the model performance. Future studies on machine learning models for the universal predicting purpose should ensure a sufficient sample size needed for complete model evaluation.”.

**3. Consideration of Confounding Factors**

The study does not fully explore perioperative factors (e.g., medication use, intraoperative hemodynamics, and surgeon expertise) that may influence pacing needs. A more detailed discussion on how the type of surgery affects pacing risk is necessary. For example, why do non-CABG procedures increase the likelihood of pacing?

A: To better explain the increased risk of pacing in non-CABG procedures, which are in fact the valvular surgery here, we rewrote the Discussion 4.3 part as following,

“Valvular surgery is acknowledged to be a risk factor of AV block and can increase the risk of temporary pacing. From an anatomy perspective, valve-related procedures often carry a risk of AV block due to its proximity of the AV conduction axis, and surgical operations can cause iatrogenic injuries[21]. Technical advancements are needed to prevent such injuries. In contrary, CABG only has operations on the large branches of coronary arteries on the surface of the heart, which hardly causes damages to the conduction system in the deep layer, less requiring pacing compared with the valvular surgery. The risk factors of AV block after CABG includes old age, CPB factors and medication[22]. The future studies may group operation types into several categories according to these features or develop specialized evaluations for high risk procedures.”,

We believe that these sentences can explain the reason through the convincing anatomical aspects.

Additionally, the anti-atrial fibrillation medication is an important confounding factor, so we added this sentence in the corresponding part

“Additionally, the influence of anti-atrial fibrillation medication should be considered as a confounding factor.”

However, considering (1) other researches also identified that atrial fibrillation can cause postoperative cardiac arrest or similar events, and (2) the mechanisms explaining why atrial fibrillation increases the events’ risk are reasonable, we do not conclude that the medication confounding can overturn our results. This is not the same case as we have between atrial fibrillation and its ablation.

The intraoperative hemodynamics can be quite vague in our topic. In the CPB assisted operations, the blood flow is in a special circulation, and we believe that the factor inclusion of CPB pump, circulation type, hypothermia, cardioplegia (volume and type) may be sufficient to indicate its statues. As far as we know, there is no study including these factors on temporary pacing. We agree a better intraoperative hemodynamic assessment is necessary, as we did not find independent risk factors within the selected parameters, while another study reported that pulmonary hypertension was a risk factor.

**4. Limitations and Bias**

Q: The manuscript acknowledges selection bias but does not sufficiently elaborate on recall bias, missing data issues, or potential inaccuracies in data collection. The retrospective design limits causal inference, which should be explicitly stated in relation to future prospective studies.

A: Although this is a retrospective study, our data were specially collected in the acknowledged clinical projects guided by corresponding methods, and therefore we believe that we minimized the recall bias, which has been emphasized in the Method 2.1 part in this revision. Usually, the missing data issues and inaccuracies in data collection can be concluded as the so called “information bias”, and they were stated in the Discussion 4.5 part with the specific sentence “information (including missing data bias caused by the update of recording systems, ascertainment bias in diagnoses, and personal factors)”. In this revision we also added the personal factors that were obviously not avoidable here. We believe that the inaccuracies in data collection were mostly the ascertainment bias in diagnoses, because although the indication of pacing was clear, it is hard to ensure fully unified diagnoses from different persons according to the ECG and cardiac output results. In the Discussion 4.5 part, we advocate having multi-center cohort studies on currently consensus factors for better causal inference, and we believe that with the new technologies developed (e.g. the millimetre-scale bioresorbable optoelectronic system for temporary pacing reported recently in Nature), this issue may be well investigated with minimized selection bias through RCT designs.

**5. Clinical Implementation**

The discussion should elaborate on how the scoring system could be integrated into clinical workflows. Exploring the potential for incorporation into electronic health records (EHRs) for automated decision support would enhance practical utility.

We added a Discussion 4.4 part to discuss the clinical implementation of our study. We found your suggestion of incorporation of EHRs enlightening, and therefore we have this sentence at the end of this part “The ideal evaluation of pacing need should be simple, effective, clinically appropriate, and incorporating local epidemiological data into electronic health records to enable automated decision support would significantly enhance clinical utility”.
